# Influence of endogamy and mitochondrial DNA on immunological parameters in cattle

**DOI:** 10.1186/1746-6148-10-79

**Published:** 2014-04-02

**Authors:** Auricélio A Macedo, Joely F F Bittar, Paula B Bassi, Juliano B Ronda, Eustáquio R Bittar, João C C Panetto, Márcio S S Araujo, Renato L Santos, Olindo A Martins-Filho

**Affiliations:** 1 Departamento de Clínica e Cirurgia Veterinárias, Escola de Veterinária, Universidade Federal de Minas Gerais, Belo Horizonte, Brazil; 2 Universidade de Uberaba, Uberaba, Minas Gerais, Brazil; 3 Centro de Pesquisas René Rachou, Fundação Oswaldo Cruz, Laboratório de Biomarcadores de Diagnóstico e Monitoração, Belo Horizonte 1715, 30190-002, Brazil

**Keywords:** Endogamy, Cattle, mtDNA, Flow cytometry

## Abstract

**Background:**

Endogamy increases the risk of manifestation of deleterious recessive genes. Mitochondrial DNA allows the separation of American Zebu (*Bos indicus* and *Bos taurus*) and evaluate the effect of mitochondrial DNA on productive traits of cattle. However, the effect of endogamy and mitochondrial DNA (mtDNA) on the immune system remains unclear. The aim of this study was to evaluate the association between endogamy, mtDNA and immune parameters.

**Results:**

A total of 86 cattle (43 cows and 43 calves) were used in this study. Age, endogamy, milk yield, and origin of mtDNA were measured and their influence on immunological parameters was evaluated. Older cows had increased CD4^+^ T cells, decreased CD21^+^ and γδ^high^ T cells as well as increased CD4^+^/CD8^+^ and T/B ratio. Multiple regression analysis indicated that endogamy in calves was associated with increased CD8^+^ T and CD21^+^ B lymphocytes, and decreased γδ^high^ T cells in peripheral blood. Cows with medium and lower endogamy had a lower percentage of B lymphocytes and γδ^low^ T cells and cows with lower endogamy had higher levels of γδ T cells and γδ^high^ T cells, as well as the CD4^+^/CD48^+^ cell ratio. Calves with higher endogamy had higher levels of CD8^+^ T lymphocytes, whereas calves with lower endogamy had lower levels of γδ^low^ T cells.

**Conclusions:**

These results demonstrated for the first time that endogamy influences the immune system of cattle.

## Background

The degree of endogamy or consanguinity in the offspring increases proportionally to the genetic similarity of its ancestors, in contrast to individuals that are randomly selected from a given population. The endogamy coefficient of an individual indicates the probability that a pair of randomly selected genes of the same locus is completely identical. Therefore, endogamic individuals can only result from mating between closely related ancestors [1,2].

The study of endogamy in cattle is extremely important, particularly due to the widespread use of artificial insemination, which allows a high selection pressure and rapid genetic gain, resulting in thousands of descendants from one single sire. Despite the unquestionable genetic gain and consequent improvement in productive traits, extensive use of a few bulls may lead to reduced genetic variability and potential deleterious effects caused by endogamy [1,3].

Endogamy decreases the frequency of heterozygotes in the population, increasing the risk of deleterious effects of recessive genes, which may negatively influence the average productive performance in the population, a phenomenon known as endogamy depression [4,5]. Therefore, the primary effect of endogamy is an increase the frequency of homozygous genes, favoring manifestation of several recessive genes, which usually cause impairment on the average individual phenotypic merit. Possible causes for declining of phenotypic value as a consequence of endogamy includes the fact that favorable genes tend to be dominant or partially dominant [6-8]. Deleterious effects of endogamy on the reproductive ability of cows and heifers have been reported [1,2,9,10]. However, whether endogamy influences the immune system remains to be investigated. Importantly, some studies report an association between low genetic diversity and increased susceptibility to diseases [8,11].

Evaluation of mitocondrial DNA allows separation of American Zebu cattle, according to its maternal lineage ancestry, into two groups: one with only *Bos taurus indicus* mtDNA (*Bi*-mtDNA) and other with *Bos taurus taurus* mtDNA (*Bt*-mtDNA) [12]. Studies to evaluate the effect of mitochondrial DNA on productive and reproductive traits in Gir and Guzerat have been conducted and show that the mitochondrial origin (*taurus* or *indicus*) significantly affects the age at first calving [13] and it does not seem to have significant effects on lactation milk yield, days in milk, or calving interval [12].

Considering the scarcity of studies regarding the effect of endogamy and mtDNA on immunological parameters, the goal of this study was to evaluate the relationship association between endogamy and the immune system. Our focus was to understand variations of immune parameters, including CD4^+^ T cells, CD8^+^ T cells, γδ T cells, and B cells (CD21^+^) in peripheral blood, that may be associated to endogamy in Guzerat (Zebu) cattle.

## Results

### Immunological parameters are influenced by endogamy

Multiple regression models including milk yield, age, mtDNA, and endogamy coefficient for cows, and mtDNA and endogamy coefficient for calves in relation to levels of lymphocyte subsets in the peripheral blood were performed and the results are summarized in Tables 1 and 2. The endogamy coefficient was significantly associated with increased CD21^+^ levels (p < 0.05; Table 1). In calves, multiple regression analysis indicated that endogamy was significantly associated with increased CD8^+^ T and CD21^+^ cells, and with significantly decreased γδ^high^ T cells and T/B cell ratio (p < 0.05; Table 2). Importantly, the age of cows was significantly associated with increased CD4^+^ T cells, CD4^+^/CD8^+^ and T/B cell ratio, and with significantly decreased CD21^+^ and γδ^high^ T cells (p < 0.05; Table 1).

**Table 1:** Multiple regression analysis of the effect of endogamy, mtDNA, age, and milk yield, on levels of lymphocytes in peripheral blood of cows*

**Lymphocytes**	**R**^**2 **^**(%)**	**Parameter**	**Coefficient**	**95% confidence interval**	**P value**
**CD4**^**+ **^**cells**	33.32	Endogamy	-0.3363	-1.001 to 0.3284	0.3120
		mtDNA	-0.6537	-5.304 to 3.996	0.7774
		*Age*	*0.003929*	*0.001868 to 0.005989*	*0.0004*
		Milk yield	0.008814	-0.003647 to 0.02128	0.1602
**CD21**^**+ **^**cells**	28.94	*Endogamy*	*0.5605*	*0.04471 to 1.076*	*0.0339*
		mtDNA	2.119	-1.489 to 5.728	0.2417
		*Age*	*-0.00181*	*-0.003409 to -0.0002116*	*0.0275*
		Milk yield	-0.00960	-0.01927 to 0.00007	0.0515
**γδ**^**high **^**T cells**	27.56	Endogamy	-0.1186	-0.3202 to 0.08306	0.2410
		mtDNA	0.6985	-0.7122 to 2.109	0.3223
		*Age*	*-0.000941*	*-0.001566 to -0.0003161*	*0.0042*
		Milk yield	0.001463	-0.002318 to 0.005243	0.4382
**γδ**^**low **^**T cells**	22.65	Endogamy	0.01741	-0.1262 to 0.1610	0.8074
		mtDNA	0.7841	-0.2208 to 1.789	0.1224
		*Age*	*0.0006863*	*0.0002410 to 0.001132*	*0.0034*
		Milk yield	0.001075	-0.001618 to 0.003769	0.4238
**CD4**^**+**^**/CD8**^**+ **^**ratio**	26.34	Endogamy	-0.01696	-0.07484 to 0.04093	0.5566
		mtDNA	-0.05224	-0.4572 to 0.3528	0.7953
		*Age*	*0.0003006*	*0.0001211 to 0.0004800*	*0.0016*
		Milk yield	0.0001360	-0.0009493 to 0.001221	0.8010
**T/B cell ratio**	36.41	Endogamy	-0.2270	-0.4592 to 0.005096	0.0549
		mtDNA	-1.311	-2.935 to 0.3127	0.1103
		*Age*	*0.001021*	*0.0003019 to 0.001741*	*0.0066*
		*Milk yield*	*0.005461*	*0.001109 to 0.009813*	*0.0153*

*Significant differences at P < 0.05 are highlighted in *italic* format. CD8^+^, and γδ T lymphocytes did show any significant correlation.

**Table 2:** Multiple regression analysis of the effect of endogamy and mtDNA on levels of lymphocytes in peripheral blood of calves*

**Lymphocyte**	**R**^**2 **^**(%)**	**Parameter**	**Coefficient**	**95% confidence interval**	**P value**
CD8^+^ cells	9.64	*Endogamy*	*0.3065*	*0.002021 to 0.6110*	*0.0486*
		mtDNA	0.4988	-3.296 to 4.294	0.7919
CD21^+^ cells	12.56	*Endogamy*	*0.4109*	*0.03578 to 0.7859*	*0.0326*
		mtDNA	-0.4894	-5.164 to 4.186	0.8335
γδ^high^ T cells	21.66	*Endogamy*	*-0.5947*	*-0.9624 to -0.2271*	*0.0022*
		mtDNA	-3.545	-8.127 to 1.038	0.1259
T/B cell ratio	11.15	*Endogamy*	*-0.09596*	*-0.1884 to -0.003520*	*0.0423*
		mtDNA	0.06902	-1.083 to 1.221	0.9042

*Significant differences at P < 0.05 are highlighted in *italic* format. CD4^+^, γδ , γδ^low^ T lymphocytes and CD4^+^/CD8^+^ cell ratio did not show any significant correlation.

Regression analysis was also performed to verify possible associations between the endogamy coefficient and immunological parameters. In cows, there were no statistically significant correlations. However, in calves there was a positive correlation between endogamy and CD8^+^ T lymphocytes (p < 0.05, r = 0.3080) and CD21^+^ B cells (p < 0.05, r = 0.3530), and a negative correlation with γδ^high^ T cells (p < 0.05, r = –0.3113) and T/B cell ratio (p < 0.05, r = –0.3334).

### Endogamy alters the immune profile of Guzerat cattle

**Figure 1:**
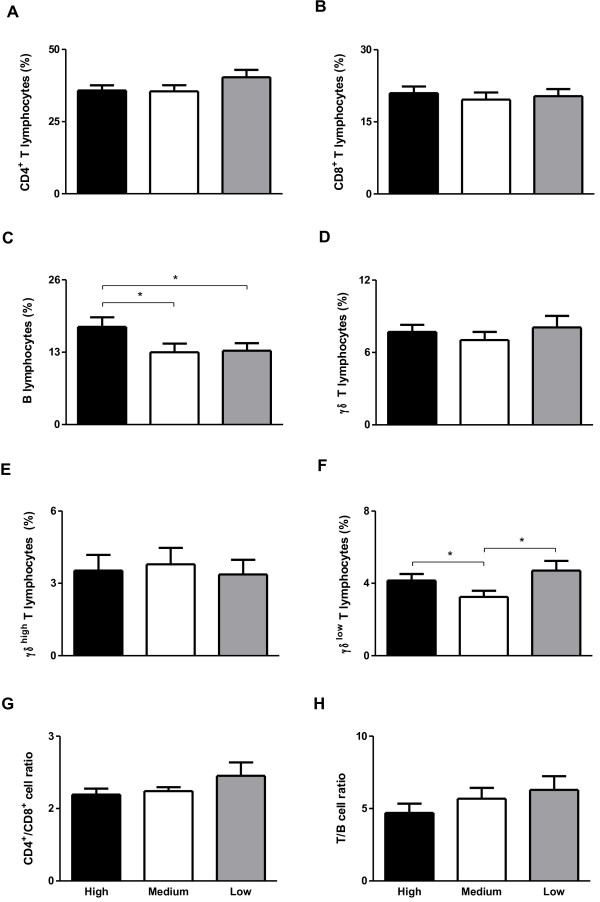
**Percentage of lymphocytes in peripheral blood from Guzerat cows with high endogamy (n = 15), medium endogamy (n = 14) or low endogamy (n = 14) analyzed by flow cytometry. (A)** CD4^+^ T lymphocytes, **(B)** CD8^+^ T lymphocytes, **(C)** B lymphocytes, **(D)** γδ^+^ T lymphocytes, **(E)** γδ^high^ T lymphocytes, **(F)** γδ^low^ T lymphocytes, **(G)** CD4^+^/CD8^+^ cell ratio and **(H)** T/B cell ratio. Data represent mean and standard error. Statistically significant differences are indicated by asterisks (* p<0.05).

**Figure 2:**
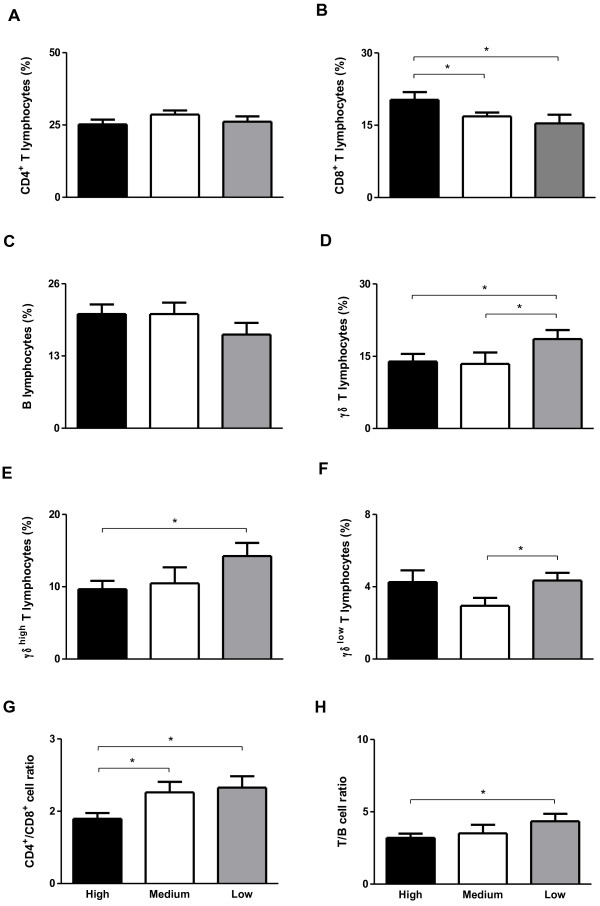
**Percentage of lymphocytes in peripheral blood from Guzerat calves with high endogamy (n = 15), medium endogamy (n = 14) or low endogamy (n = 14) analyzed by flow cytometry. (A)** CD4^+^ T lymphocytes, **(B)** CD8^+^ T lymphocytes, **(C)** B lymphocytes, **(D)** γδ^+^ T lymphocytes, **(E)** γδ^high^ T lymphocytes, **(F)** γδ^low^ T lymphocytes, **(G)** CD4^+^/CD8^+^ cell ratio and **(H)** T/B cell ratio. Data represent mean and standard error. Statistically significant differences are indicated by asterisks (* p<0.05).

Based on endogamy coefficients, cows and calves were grouped into high, medium, and low endogamy as described in Methods. The immune profiles of these cattle were then analyzed by flow cytometry (Figures 1 and 2). These results indicated that cows with medium and low endogamy had a decreased percentage of B lymphocytes when compared to cows with high endogamy (p < 0.05; Figure 1C). Furthermore, cows with lower endogamy had a higher percentage of γδ^low^ T cells when compared with cows with medium endogamy (p < 0.05; Figure 1F). There was also a clear influence of endogamy on the immunological profile of calves. Calves with high endogamy had an increased percentage of CD8^+^ T lymphocytes when compared to calves with medium and low endogamy (p < 0.05, Figure 2B). In contrast, calves with low endogamy had an increased percentage of γδT cells (p < 0.05; Figure 2D) and γδ^high^ T cells (p < 0.05; Figure 2E) as well as the CD4^+^/CD8^+^ T cell ratio (p < 0.05; Figure 2G) and T/B ratio (p < 0.05; Figure 2H) when compared to calves with high endogamy (p < 0.05). Calves with low endogamy had a decreased percentage of γδ^low^ T cells only in comparison to those with medium endogamy (p < 0.05; Figure 2F).

### European mtDNA in Guzerat is associated with low endogamy and differences in immunological parameters

**Figure 3:**
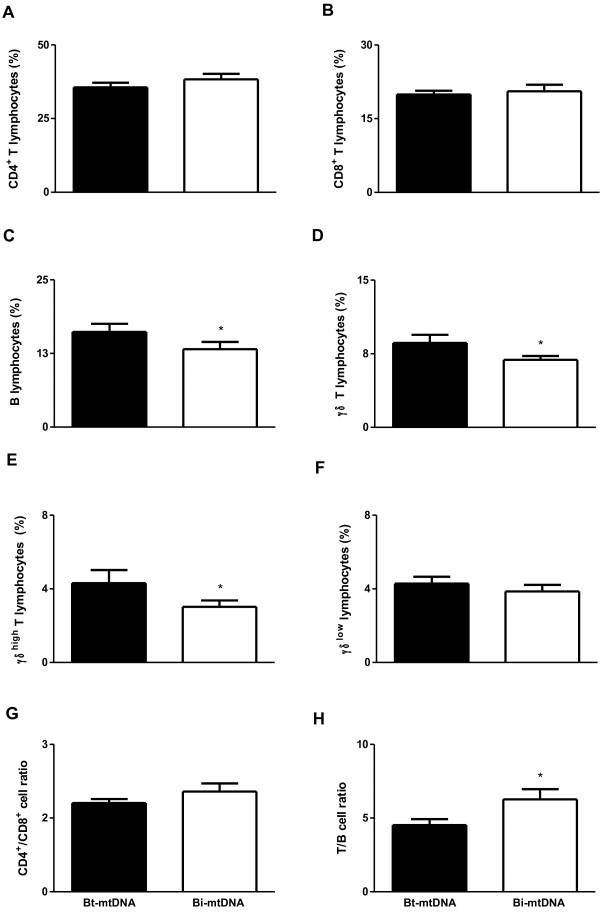
**Percentage of lymphocytes as assessed by flow cytometry in peripheral blood from Guzerat cows either with *Bt-*mtDNA (n = 18) sequences or with *Bi-*mtDNA (n = 25). (A)** CD4^+^ T lymphocytes, **(B)** CD8^+^ T lymphocytes, **(C)** B lymphocytes, **(D)** γδ^+^ T lymphocytes, **(E)** γδ^high^ T lymphocytes, **(F)** γδ^low^ T lymphocytes, **(G)** CD4^+^/CD8^+^ cell ratio and **(H)** T/B cell ratio. Data represent mean and standard error. Statistically significant differences are indicated by asterisks (* p<0.05).

**Figure 4:**
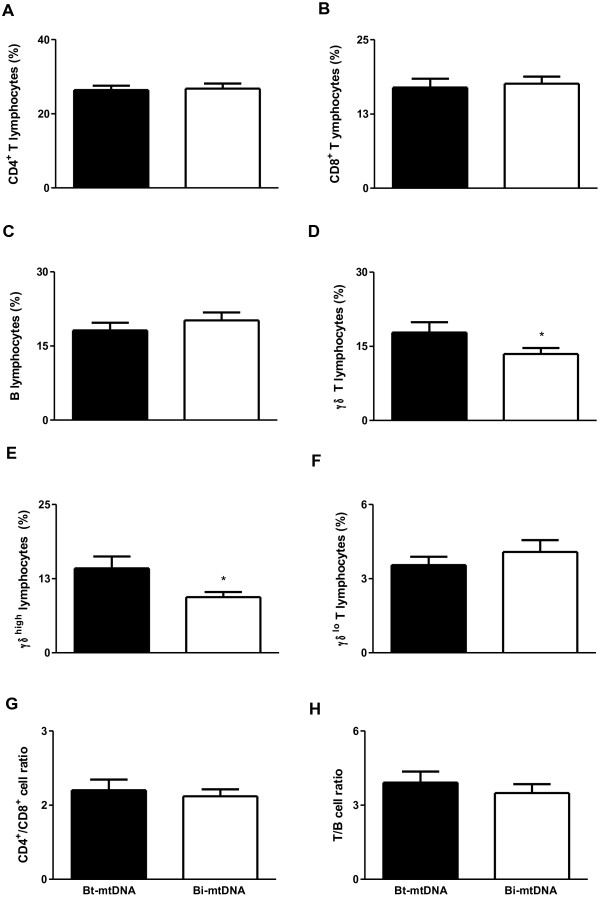
**Percentage of lymphocytes as assessed by flow cytometry in peripheral blood from Guzerat calves either with *Bt-*mtDNA (n = 18) sequences or with *Bi-*mtDNA (n = 25). (A)** CD4^+^ T lymphocytes, **(B)** CD8^+^ T lymphocytes, **(C)** B lymphocytes, **(D)** γδ^+^ T lymphocytes, **(E)** γδ^high^ T lymphocytes, **(F)** γδ^low^ T lymphocytes, **(G)** CD4^+^/CD8^+^ cell ratio and **(H)** T/B cell ratio. Data represent mean and standard error. Statistically significant differences are indicated by asterisks (* p<0.05).

**Figure 5:**
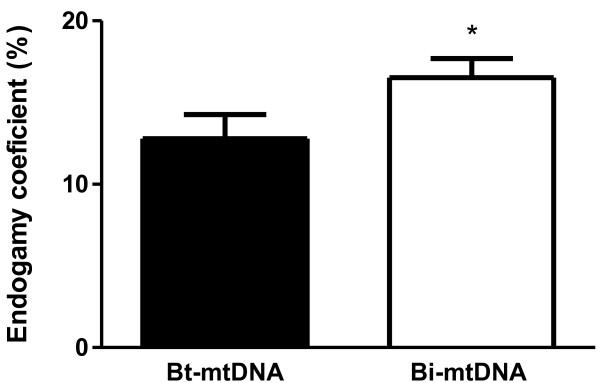
**Endogamy coefficient in Guzerat calves either with *Bt-*mtDNA (n = 18) sequences or with *Bi-*mtDNA (n = 25).** Data represent mean and standard error. Statistically significant differences are indicated by asterisks (* p < 0.05).

All cattle used in this study were mtDNA genotyped. This analysis allowed us to assess whether individual Zebu cattle (*Bos taurus indicus*) remained with no introduction of maternal DNA of *B. taurus taurus* origin, thus tending to have higher levels of endogamy [12]. Our results demonstrated that 42% (18/43) of the cows had mtDNA from *B. taurus taurus*, and 58% (25/43) had only *B. taurus indicus* (Zebu) mtDNA. The same result was observed in calves, which was expected since these calves were the offspring of those same cows mentioned above. After grouping cows and calves according to the presence or absence of *B. taurus taurus* (European) mtDNA, which were classified into “*Bt-*mtDNA” or “*Bi-*mtDNA”, respectively, the profile of leukocytes in peripheral blood was analyzed by flow cytometry. In cows, the percentages of B lymphocytes, γδT cells, and γδ^high^ T cells were decreased in individuals with *Bi-*mtDNA when compared to individuals with *Bt-*mtDNA (p < 0.05; Figure 3C, D, and E, respectively). In calves, individuals with *Bi-*mtDNA had a decreased percentage of γδT cells and γδ^high^ T cells when compared to individuals with *Bt-*mtDNA (p < 0.05; Figure 4D and E, respectively). As expected, the endogamy coefficient was lower in calves with *Bt-*mtDNAwhen compared to calves with *Bi-*mtDNA (p < 0.05; Figure 5).

## Discussion

Here we demonstrated a clear effect of endogamy on immune parameters of Guzerat cattle. This is the first evidence that endogamy may affect the immunological parameters in cattle. The main consequences of endogamy increase are the reduction of genetic variability within inbred lines and increased frequency of homozygosity, which may be desirable for alleles with favorable effects. However, a small fraction of undesirable deleterious recessive alleles may have an enhancement of their manifestation under these conditions. Therefore, endogamy tends to increase expression of undesirable recessive alleles [14].

Deleterious effects of endogamy have been demonstrated, especially on reproductive traits [1,2,9,10]. A study of inbreed and outbreed populations of several different species of captive ungulates indicates that the rate of neonatal mortality is higher in endogamic when compared to non endogamic animals [15]. In humans, consanguinity is an important risk factor for susceptibility to infectious diseases [16]. However, direct associations of endogamy with the immune system have not been previously investigated in animals.

Higher levels of endogamy clearly affected some of the immunological parameters, particularly γδ T cells. These cells are the major circulating lymphocyte subset population in ruminants, especially in calves. These cells play a role in the transition between innate and adaptive immune [17]. γδ T lymphocytes can recognize and be activated by pathogen-associated molecular patterns (PAMPs) or danger-associated molecular patterns (DAMPs) in absence of other stimuli [18,19], supporting the notion of an innate immune function of these cells. However, other studies demonstrated an important role of γδ T lymphocytes in adaptive response since they recognize antigens presented on the surface of antigen-presenting cells (APCs), and they have cytotoxic effect mediated by expression of granulysin [20] and perforin [19,21]. These cells play an important role during infection with *Mycobacterium bovis*, the causative agent of bovine tuberculosis [21-23]. Recent studies have also demonstrated the role of γδ T lymphocytes as APCs [23-25] so they may play a role in developing protective immune responses following vaccination [17], and are important for host defense during *Brucella abortus* infection, when these cells enhance macrophage function via IFN-γ [26].

Although resistance or susceptibility to disease involves multifactorial mechanisms, it has been reported that Zebu cattle tend to have a higher degree of natural resistance to diseases when compared to European cattle [27-31]. Analyzing the profile of peripheral blood leukocytes of different bovine breeds, we recently demonstrated that Guzerat cattle comparatively has increased levels of circulating leukocytes, especially monocytes, eosinophils, T lymphocytes (particularly CD4^+^ T cells) and non-B/non-T (NTNB) lymphocytes, with a decreased percentage of B lymphocytes, and consequent increase in the T/B and CD4^+^/CD8^+^ ratio [29]. In another study, we compared the macrophage function of Zebu cattle with that of macrophages from European cattle. Macrophages from Zebu are more efficient in controlling intracellular replication of *Brucella abortus*. Furthermore, Zebu macrophages generate higher levels of nitric oxide and cytokines such as IL-12 and TNF-α [30]. Therefore, increasing lines of evidences support the notion that resistance of Zebu cattle may be due to quantitative and qualitative differences in immune cells. In this study, we addressed for the first time whether endogamy have any influence on the profile of immune cells in the peripheral blood of Zebu cattle.

Here we demonstrated that age affected most of the immunological parameters of cows. The influence of age on the immune system has already been demonstrated in both animals and humans, affecting both innate and adaptive immune systems [32-35]. Age-related decrease in B cell activity, potentially related to the decline in helper T cell activity, and decreased cytolytic and delayed-type hypersensitivity responses have been described [32,36]. The age-related alteration in helper T cell activity appears to be due to intrinsic or functional changes within the cells, as well as to a shift in subset proportions of CD4^+^ and CD8^+^ T cell populations, with increases in the memory phenotype and corresponding decreases in the naive phenotype [37].

## Conclusions

In conclusion, our data indicate that endogamy influences the immune system of cattle since it is associated with significant changes in the profile of immune cells in the peripheral blood, particularly decreased levels of γδ T lymphocytes, an important subpopulation of lymphocytes in cattle, especially in calves.

## Methods

### Cattle and samples

A total of 43 Guzerat (*Bos taurus indicus*) cows with an average of 8 (3–17) years of age and their progeny (a total of 43 male or female calves) with an average of 12 (2.4–25) month-old were included in this study. The average daily milk yield of cows was 18.4 (4.4–31) liters per day. These cattle were maintained under regular management conditions in their farm. They were free of ticks and hemoparasites. Blood samples were collected within 5 mL tubes containing ethylene diamine tetra acetic acid (EDTA) for *ex vivo* cellular evaluation, and stored at room temperature until further processing. This study was approved by the Ethical Committee for the use of Experimental Animals of the Universidade Federal de Minas Gerais, Brazil (CETEA), under protocol 139/2010.

### Analysis of endogamy coefficient

All cattle included in this study were subjected to artificial insemination with a highly stringent breeding control, and have registered *Bos indicus* breed Guzerat pedigrees since 1895 (over 100 years) that guarantee a very low risk of pedigree error. The individual inbreeding coefficients were calculated by inverting the diagonal of the inverse matrix of kinship using the set of multiple-trait derivative-free - MTDFREML software package [38]. The software calculates the inverse of the relationship matrix directly from a list of animals and their parents, providing individual identification for matching phenotypic records to individuals and calculating the inbreeding coefficients as well as the logarithm of the determinant of the relationship matrix needed to calculate the logarithm of the likelihood function. The program also prepares the coefficients for the mixed model equations based on the statistical model for single and multiple trait analyses. Then, the program solves the mixed model equations and finds variance component estimates that maximize the restricted likelihood given the phenotypic data. Based on the inbreeding coefficients, the animals were grouped at low, medium and high endogamy. Endogamy coefficients were first ordered from the highest to the lowest and the upper third was considered high endogamy (coefficients ranging from 26.4 to 16.3, and 26.1 to 18.1, for cows and calves, respectively), the mid third was considered medium endogamy (coefficients ranging from 16.2 to 14.2, and 18.0 to 15.4, for cows and calves, respectively), and the lower third was considered low endogamy (coefficients ranging from 14.0 to 9.8, and 15.3 to 0.0, for cows and calves, respectively).

### Mitochondrial DNA analysis

Analysis of mitochondrial DNA (mtDNA) was performed as previously described [12]. Polymorphisms for *B. indicus* and *B. taurus* mtDNA were identified using two other RFLP markers. Briefly, total DNA samples from several animals were re-amplified by PCR using primers 5’-CCCAACGAGGAAAATATACC-3’ (BosmtF1) and 5’-AACCGCAAACAACCTCTTCC-3’ (BosmtR1) targeting a region of the ND5 gene of the mitochondrial genome. Amplified mtDNA was then digested *Hin*dIII for 1 h at 37°C, electrophoresed on 1.5% agarose gel, stained with ethidium bromide and evaluated with a Fuji Fla 3000G Laser Scanner (Fuji Film Co., Tokyo, Japan), using Image Gauge version 3.12 software. The presence of the HindIII restriction site within the amplified region is indicative of B. taurus taurus mtDNA. Efficiency of HindIII digestion was assured by the complete absence of partially digested fragments.

### Immunophenotyping of peripheral lymphocytes

Immunophenotyping was performed using specific monoclonal anti-bovine cell receptor antibodies as previously described [29]. Briefly, these antibodies were tittered, and 1:15, 1:30, 1:60, and 1:120 dilutions were used for anti-CD4, anti-CD8, γδ T and anti-CD21 antibodies, respectively. A single cell type was analyzed in each tube. After incubation with primary antibodies, erythrocytes were lysed by incubating with 2 mL lysis solution (FACS™ Lysing Solution – BD Biosciences, San Jose, CA, USA) for 8 min at room temperature. After lysis, leukocytes were washed in PBS and fixed with 200 μL Max FaxFix. Acquisition, storage, and analysis of the data were performed using a FACScalibur flow cytometer (BD Biosciences, San Jose, CA, USA) with CellQuest software (BD Biosciences, San Jose, CA, USA). Leukocyte subpopulations were quantified based on size (laser forward scatter; FSC) and granularity. Lymphocyte subsets were analyzed considering cells within the selected lymphocyte region based on the relative fluorescence intensity observed in the FL1 versus FL2 dot plot distributions. Quadrant statistical analysis was applied to quantify the fluorescent positive lymphocyte subset within the lower right quadrant. Calculations of the CD4^+^/CD8^+^ T cell and T/B cell ratio and percentage of γδ^high^ T andγδ^low^ T cells (based on the expression profile among the total population of γδ T cells) were also performed.

### Statistical analysis

Several parameters including age, milk yield, mtDNA, and the endogamy coefficient were considered in these analyses. Multiple regression analyzes and linear correlation were performed using the Graphpad Instat3 software (GraphPad Software, San Diego, CA, USA).

Flow cytometric data were submitted to analysis of variance and means were compared by the Student-Newman-Keuls test (SNK) using the Prisma Graphpad 5.0 software (GraphPad Software, San Diego, CA, USA). Data were considered statistically different when p < 0.05.

## Competing interests

The authors confirm that they have no conflicts of interest in this work.

## Authors’ contributions

AAM and PBB designed and prepared the manuscript. JFFB, JBR, ERB and JCCP performed the experiments. MSSA, RLS and OAMF supervised the study. All authors have read and approved the final manuscript.
